# A Review of Processing, Marketing, and Demographic Influence on Consumer Perception of Pulse Milk‐ Insights From Traditional and AI‐Facilitated Research

**DOI:** 10.1111/1750-3841.70403

**Published:** 2025-07-14

**Authors:** Chidimma Ifeh, Bradley Whitaker, Neda Nazemi, Mark Greenwood, Mary Miles, Wan‐Yuan Kuo

**Affiliations:** ^1^ Department of Food Systems, Nutrition and Kinesiology Montana State University Bozeman Montana USA; ^2^ Department of Electrical and Computer Engineering Montana State University Bozeman Montana USA; ^3^ Department of Computer Science Montana State University Bozeman Montana USA; ^4^ Department of Mathematical Sciences Montana State University Bozeman Montana USA

**Keywords:** artificial intelligence, plant‐based milk, product development, sensory, sustainability

## Abstract

Sustainable, healthy, and environmental concerns are part of the reasons consumers are shifting to plant‐based diets, such as plant‐based milk, which has reduced greenhouse gas emissions and water footprint as an advantage over animal‐based diets. Pulse crops such as lentils, lupins, chickpeas, and peas offer nutritious and health benefits, containing proteins, dietary fiber, and low‐fat content, as well as soil health restoration and cropping diversification as environmental benefits. Pulse milk is gaining popularity as one of the broader plant‐based milk categories that offer beneficial dairy alternatives. This review summarizes the impact of processing, marketing, and demographic variables and how they influence consumer perception of pulse milk. We also highlighted insights from artificial intelligence (AI) and traditional research methods to understand consumer choices. The consumer acceptance of pulses faces some challenges despite their benefits, related to sensory attributes such as beany, earthy, and grassy off‐flavors and the lack of culinary knowledge amongst consumers. The lower government subsidies for pulses compared to common crops and the lack of sustainability benefits awareness by some consumers limit pulse crop production. Advanced processing techniques like enzymatic treatments and homogenization can enhance pulse milk sensory quality. Marketing strategies tailored to target different demographics can encourage consumption. When developing a food product, analyzing relationships becomes difficult when there are multi‐faceted variables to consider. The gap in traditional research methods can be bridged with AI‐facilitated methods in predicting consumer preferences in product development. Further research is needed to enhance the nutritional and sensory quality of pulse milk.

## 1 Introduction

More consumers are limiting their consumption of animal‐based diets and shifting to plant‐based diets due to health concerns, greenhouse gas emissions, and animal welfare issues associated with industrial livestock farming (Jeske et al. 2019; Kumar et al. 2023). The weekly average of cow milk purchased by American households at retail food stores in 2017 declined by 12%, while the number of plant‐based milk purchased per household increased by 33% (Stewart et al. 2020). Pulse milk is part of the broader plant‐based milk category gaining popularity as a valuable dairy substitute as consumers shift to plant‐based diets. The global pulse‐based food market has an estimated worth of 78.0 billion US dollars in 2024, with a projected 5.7% annual growth to 135.2 billion by 2034 (Future Market Insights 2024).

Pulses belong to the family *Leguminosae* and are crops such as lentils, lupin, chickpeas, and peas grown for their dry seeds (FAO 2024). Pulses are nutritious, with about 21 to 25% protein, a good source of carbohydrates, dietary fiber, minerals, vitamins, and phytochemicals (Singh 2017). The dietary incorporation of pulses is beneficial for improving digestive health and reducing low‐grade inflammation and the risk of chronic diseases (Mullins and Arjmandi 2021). The nutritional qualities of pulses make them an excellent choice for a balanced and health‐conscious diet. The production of pulse crops has a lower environmental impact than dairy farming (Craig et al. 2023), as the cultivation of pulses can improve soil nutrients and moisture while reducing nitrogenous greenhouse gas emissions (Hossain et al. 2016).

About 2.6 million pounds of pulses are harvested each year by United States farmers, with over 75% being exported (Asif et al. 2013). Pulses are underutilized crops in the Western world, considering that just 7.9% to 13.1% of North Americans eat pulses on any given day (Mudryj et al. 2014). The lower government subsidies for pulses compared to other common crops such as wheat and corn, and the lack of sustainability benefits awareness by some consumers, limits pulse crop production by farmers (Dhazzar 2024). In the United States, assistance like the Government Emergency Commodity Assistance Program (ECAP) offers higher payment rates to other crops like rice ($76.94/acre) and oats ($77.66/acre), than pulse crops like lentils ($19.30/acre) and peas ($16.02/acre) (USDA 2025). Such economic assistance to lessen the effects of rising input costs and declining commodity prices could make other crops financially attractive despite the sustainability benefits of pulse crops. The utilization and market potential of value‐added products from pulses, such as pulse milk, can increase with the growing trend of a plant‐based diet. Roland et al. (2017) studies on the inherent off‐flavor in pulses, such as beany, earthy, and grassy, can be attributed to the reason pulses are being less explored and consumed. The production of pulse milk that can provide distinct flavors potentially appealing to consumers can be developed from lentils, lupins, chickpeas, and peas. Combining different processing techniques and tailoring marketing strategies to diverse demographic preferences can enhance pulse milk sensory attributes and consumer appeal.

Processing techniques like soaking, dehulling, germination, and pressure cooking have been reported to reduce the beany flavor in pulses (Lopes et al. 2020; Andaç et al. 2024; Cichońska et al. 2024). A combination of advanced processing techniques can enhance sensory quality and consumer preference for pulse milk. Different demographics should be considered in the consumer segment (Dwivedi et al. 2021); hence, to increase consumer acceptance of pulse milk, marketing can be tailored to target demographics. Processing, marketing, and demographic factors are multi‐faced variables that can be considered when developing a food product, making the traditional research approach aiming to capture the relationships between these variables challenging to analyze.

Artificial intelligence (AI) is a tool that can be used to model large datasets to draw patterns showing links between different variables. Thus, AI can be used to create models based on labeled datasets that can predict optimal product variables, skipping through labor and time‐intensive trials (Ikram et al. 2024). Machine learning, as a subfield of AI, includes algorithmic models that learn from datasets to predict outcomes from input variables, enabling pattern recognition and decision making (Tseng et al. 2023). Bhavsar et al. (2023) developed a machine learning model to predict the quality of dairy milk using different milk parameters such as color. However, integrating AI in consumer research on pulse milk is still lacking.

**FIGURE 1:**
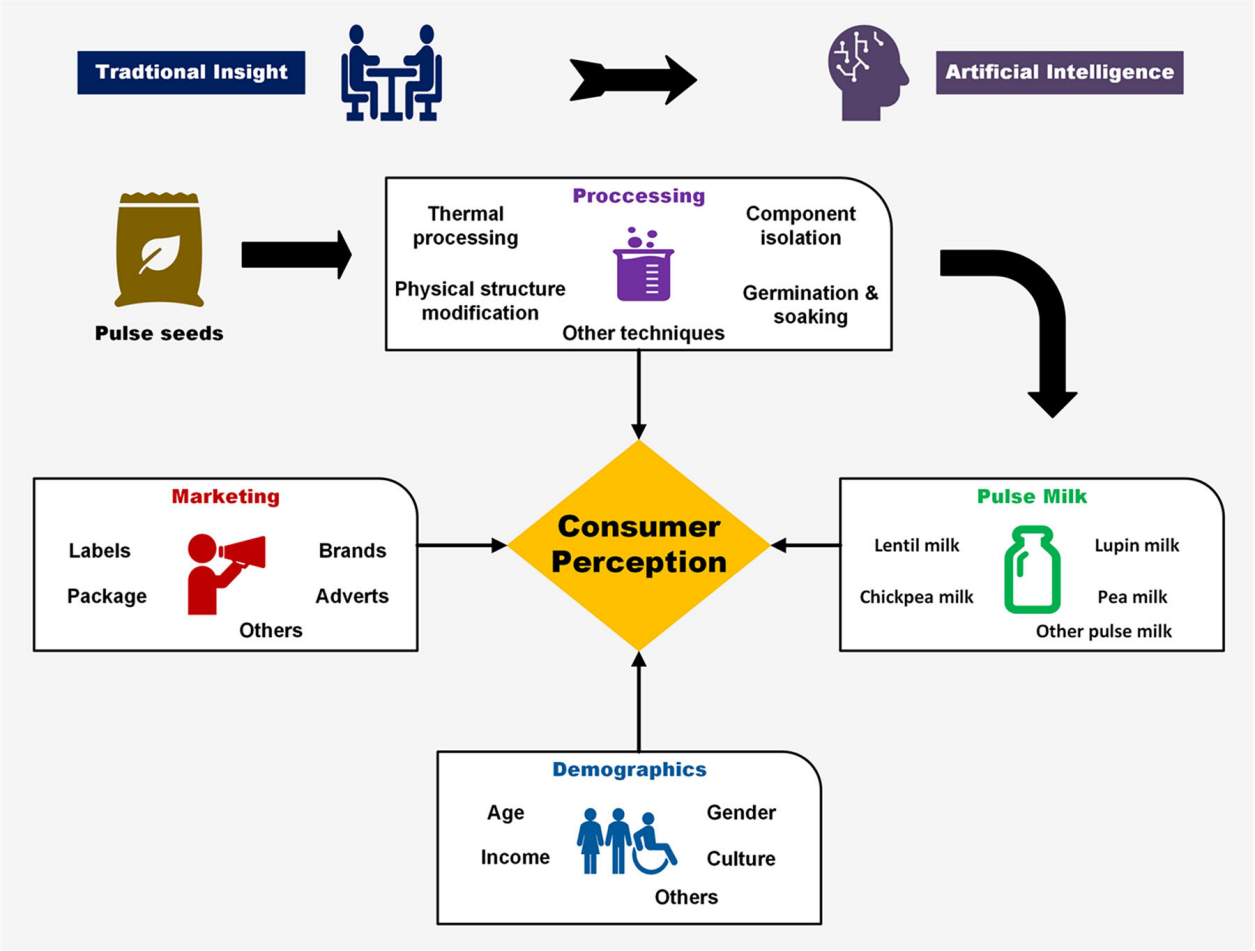
Factors influencing consumer perception of pulse milk.

The potential of pulse milk, such as lentil milk, lupin milk, chickpea milk, and pea milk, has been explored by several studies (Jeske et al. 2019; Vogelsang‐O'Dwyer et al. 2021; Zhang et al. 2022; Andaç et al. 2024). However, there is a lack of published reviews that explored the potential of AI applications to bridge traditional research methods in integrating different factors on consumer perception of pulse milk. This review discusses the influence of processing, marketing, and demographic variables on consumer perception of pulse milk (Figure 1). It further highlights insights from traditional research methods and emerging AI‐driven approaches to understanding consumer choices.

## 2 Pulses and Pulse Milk

### 2.1 Nutritional Profile of Pulses

Pulses are dry edible seeds belonging to the Fabaceae family (Kumar and Pandey 2020). Common pulses consumed worldwide include dry beans, dry chickpeas, lentils, dry peas, cowpeas, pigeon peas, Bambara groundnut, and lupins (Hall et al. 2017). The Food and Agriculture Organization (FAO) of the United Nations describes green chickpeas, green beans, and green peas as crops harvested fresh and are considered as vegetables. Not all legumes are pulses; legumes such as soybeans and peanuts are grown for oil extraction, whereas pulses are naturally dried and primarily grown for their seeds (Asif et al. 2013).

Pulses have high nutritional quality, containing dietary fiber, starch, protein, vitamins, and minerals (Hall et al. 2017). Carbohydrates are the highest component of pulses, with about 50% to 65%, containing starch (22—45%) and dietary fiber (4.3—25%), followed by a rich protein content of 19% to 30% (Zhou et al. 2024). De Almeida Costa et al. (2006) reported an animal protein content of 18% to 25% in meat. Pulses contain a low fat content of less than 3%, as reported by Hall et al. (2017). However, chickpeas have a higher fat content of approximately 6% (USDA 2023). The nutritional profiles of pulses have also been summarized and tabulated in published review papers (Carbas et al. 2023; Verma et al. 2025). Pulses are a source of minerals and vitamins, although they also contain antinutritional components such as oxalate that can lower the bioavailability of minerals (Hall et al. 2017; Zhou et al. 2024). Antinutritional compounds found in pulse seeds, such as tannins and phytic acid, are associated with pulse protein's resistance to proteolysis (Sozer et al. 2017).

Pulse consumption has been linked to other health benefits for the human body. Pulses are known to have a lower glycemic index (GI) than cereals as they contain resistant starch that is more slowly absorbed in the body (Zhou et al. 2024). Hou et al. (2023) reported that pulses contain bioactive phytochemical compounds that can reduce the risk associated with metabolic and some chronic diseases such as cancers, diabetes, and obesity. Phytochemicals are naturally occurring secondary metabolites that have important biological activities and are responsible for the organoleptic quality of food (Zhang et al. 2018). Antinutritional substances such as tannins, when taken in moderation and under certain conditions, can have therapeutic uses, such as supporting the body's defense against harmful free radicals (Singh et al. 2023). Studies have shown that tannins and phenolic acid are phytochemicals associated with antioxidant activity, gastrointestinal functions, and anti‐inflammatory properties (Alkaltham et al. 2022; Carbas et al. 2023). Carotenoids and flavonoids are phytochemicals that usually contribute to food products' color and organoleptic properties (Zhang et al. 2018). Jukanti et al. (2012) reported that pulses contain resistant starch and the highest concentration of oligosaccharides among other crops, as carbohydrates are their primary component. Oligosaccharides, though linked to prebiotic effects, also cause bloating and abdominal pain as they are not hydrolyzed by the digestive system of humans, causing flatulence and rapid gas production (Jukanti et al. 2012; Mutuyemungu et al. 2023).

### 2.2 Importance of Pulses in Food Systems Sustainability

The global human population is projected to increase from 8.0 to 9.7 billion by 2050 (United Nations 2022). It is essential to embed sustainable practices to protect our food systems, preserve resources, and mitigate the environmental impact of increased food production (FAO 2018). The demand for food, arable land, and clean water to sustain a healthy life will grow with the rise in the global population (Tian et al. 2016). Pulses directly support the planet, people, and profit, known as the sustainability three P's, by addressing food insecurity and hunger, reducing environmental degradation and the impact of climate change, and providing resilient and cost‐effective farming alternatives (Xipsiti et al. 2017; Didinger and Thompson 2020).

The cultivation of pulse crops has numerous benefits, such as enhanced soil fertility and minimal water usage (Venkidasamy et al. 2019), cropping systems diversification (Didinger and Thompson 2020), and soil health restoration (Kumar et al. 2023), while reducing nitrogenous greenhouse gas emissions (Hossain et al. 2016). The demand for pulses needs to increase for more farmers to show interest and tap into these benefits. The processing of pulses into new products such as snacks and milk can increase pulse crop utilization, offering modern convenience food (Cichońska et al. 2023). Didinger and Thompson (2020) argued that the consumption of pulses could increase if they are made delicious, convenient, and an affordable option. The study also mentioned that pulses provide a variety, such as refrigerated pre‐cooked pulses, canned pulses, as well as dry and shelf‐stable pulses that do not lose nutritional value over a long period. Pulses can serve as an essential ingredient in the food industry and can be cooked and used in soups and stews.

The EAT‐Lancet Commission designed the universal healthy diet, emphasizing the shift to plant‐based diets relying on pulses, which can nurture environmental and human health (Didinger and Thompson 2020). Magrini et al. (2018) reported that studies have shown no economic gain or loss on pulse crop introduction in the cropping systems for farmers in France. The study also mentioned that the marginalization of pulses, despite their environmental benefits, is because of cereal crops increasing returns. Hence, the production of pulse crops by farmers and pulse value‐added processing in the food industry would need private and public support for a transition to increase pulse consumption. Ullah et al. (2020) reported that barriers such as marketing issues, cultivation on marginal soils as opposed to fertile lands, lack of seed distribution, and crop improvement as the reasons for low pulse farming and crop yield in Pakistan. The study also stated that pulses are substituted for high‐yielding cereals with government subsidies that offer better economic returns and labor‐intensive practices despite their importance in sustainable food systems.

### 2.3 Types of Pulse Milk and Their Characteristics

Pulses have earthy, beany, and grassy off‐flavors, which are among the most common challenges in exploring pulse milk. Individuals explore pulse milk as an alternative to dairy milk for different reasons, such as dietary preferences and environmental and health concerns. Different pulse crops, such as chickpeas, lentils, dry peas, and lupins (FAO 2024), can be used for potential milk production. This review focuses on pea and chickpea milk, which are commercially available pulse milk, and lentils and lupins, which have the potential to be developed for the commercial market. The production of pulse milk is similar amongst the different pulse crops, with slight modifications depending on the pulse type. These include soaking, germination, thermal processing, physical structure modification, component isolation, and separation (Patterson et al. 2017; Vogelsang‐O'Dwyer et al. 2021; Zhang et al. 2022; Kulczyk et al. 2023).

Pea milk, made from pea seeds (*Pisum sativum L*.), is characterized by its low allergenicity and glycemic index and is the most common pulse milk (Asif et al. 2013). Unlike lentil and lupin milk, unsweetened pea milk is commercially available and is marketed as having 50% calcium and half the sugar content of dairy milk (Ripple Foods 2025). Commercially available pea milk brands include Ripple, Sproud, and Mighty. Pea milk is stabilized with guar gum and pectin during processing to achieve a texture and mouthfeel similar to dairy milk and ensure a creamy and homogenous appearance (Kulczyk et al. 2023). Studies involving trained panelists on sensory evaluation of pulse milk have described pea milk as having grassy, milk‐like, and earthy as the most common attributes (Andaç et al. 2024). Roland et al. (2017) reported that the off‐flavor and bitterness of peas, which is also tasted in pea milk, have been associated with saponins, volatile and non‐volatile compounds.

Chickpea milk, made from chickpea seeds (*Cicer arietinum L*.) has a protein content ranging from 1.0% to 1.5% (w/v), which is higher than that of most nut‐based milk but less than that of cow milk, which ranges from 3.0% to 3.5% (w/v) (Lopes et al. 2020). The milk from processed chickpeas is bright in appearance and has a smooth texture that could enhance its market appeal (Kishor 2017). Chickpea milk is nutritious, contains protein and unsaturated fat, is cholesterol‐free, and contributes to antioxidant and anti‐inflammatory properties (Duarte et al. 2022). Value‐added products, such as fortified beverages and yogurt from chickpea milk as a base, have achieved desirable gel‐like structures and flow properties comparable to traditional yogurts (Aguilar‐Raymundo and Vélez‐Ruiz 2019).

The nutrient‐dense seeds of lentils (*Lens culinaris*) are high in protein content and several essential amino acids (Hanley et al. 2024), making them a potential option for making lentil milk. Lentils contain between 17% and 29% protein (Nosworthy et al. 2017) and are highly digestible (∼83%) (Jeske et al. 2019), making them a good choice for pulse milk production. However, there is still no commercial‐based lentil milk despite its nutritional content, making consumers unfamiliar with the product, unlike pea milk, which has seen high marketing by available brands. Lentil contains about 63% carbohydrate content (Dhull et al. 2023), so achieving a creamy consistency similar to dairy milk and the off‐flavor is one of the challenges in commercializing lentil milk (Syamala et al. 2024), which needs advanced processing techniques. Lentil milk can come in different shades of color depending on the variety of lentil seeds used in the production. Although lentil milk has distinct variations in taste and texture compared to dairy milk, it is a sustainable alternative for consumers seeking plant‐based protein sources. Lentil milk has some potential, as it can be used as a base in making other food products, such as lentil milk‐based cheese (Moradi et al. 2021; Naeem et al. 2024) and lentil milk‐based yogurt (Verni et al. 2020; Boeck et al. 2022).

One of the least common types of pulse milk is lupin milk, made from lupin seeds (*Lupinus albus L*.). Lupins for Life, an Australian company, produces lupin‐based products like flakes, flour, crumb, and kibble; however, commercial lupin milk is not currently available in the market (Lupins for Life 2024). The nutritional value of plant‐based milk varies and can depend on processing techniques, raw materials, and fortification (Mäkinen et al. 2016). Jiménez‐Martínez et al. (2003) reported preparing lupin milk with a protein concentration of 5.8%, compared to 2.6% and 3.9% protein found in cow and soy milk, respectively. Lopes et al. (2020) reported that the most challenging factor in consumer acceptance of lupin milk was the off‐flavor, which was reduced using heat treatment. Lupin milk contains α‐galactosidase, which can aid fermentation in creating value‐added products like cheese without lactose, benefiting lactose‐intolerant individuals (Al‐Saedi et al. 2021). Sanz et al. (2010) reported that lupins contain hidden allergens recorded to trigger reactions in sensitive individuals. The allergenic property of lupin could further hinder the potential of commercializing lupin milk.

## 3 Impact of Processing Factors on Pulse Milk Quality and Consumer Perception

### 3.1 Processing Techniques Used for Pulse Milk Production and Impact on Sensory Attributes

To meet consumer demands for pulse milk, studies have applied one or more processing techniques (Table 1): soaking, germination, blanching, cooking, pasteurization, sterilizing, milling, filtration, homogenization, and stabilizing (Patterson et al. 2017; Vogelsang‐O'Dwyer et al. 2021; Zhang et al. 2022; Kulczyk et al. 2023).

**TABLE 1:** Impact of different processing techniques on pulse milk.

Processing technique on pulse milk	Lentil milk	Chickpea milk	Pea milk	Lupin milk
**Soaking and germination**	Germination at 25°C for 72 h activated hydrolytic enzymes and reduced the levels of some of the oligosaccharides in the product (Cichońska et al. 2023). Germination at 25°C for 72 h enhanced the nutritional profile by increasing the dietary fiber and levels of B vitamins in the product and decreasing starch odors (Cichońska et al. 2024)	Soaking for about 16 h effectively reduced α‐oligosaccharides, commonly known as flatulence sugars (Duarte et al. 2022). Germinating the seeds for about three days enhanced the dissolution of sugars due to carbohydrate hydrolysis, leading to a higher opacity in the milk (Lopes et al. 2020).	Soaking in alkaline water (pH = 9) for 1 h, blanching, and dehulling may have contributed to the concentration of some volatile compounds, leading to off flavors in the milk (Andaç et al. 2024).	Soaking for about 16 h decreased bitterness and germinating for about three days reduced the gelling of the milk due to starch hydrolysis (Lopes et al. 2020).
**Thermal processing**	Sterilization at 121°C for 15 min extended the shelf life, ensuring microbial safety by eliminating pathogenic and spoilage microorganisms of the product (Cichońska et al. 2023).	Cooking the seeds at 100°C for 30 min minimized the presence of unpleasant volatile compounds while reducing the pleasant odors in the original aromatic matrix (Duarte et al. 2022).	Vacuum pretreatment (0.08 MPa) of the seeds at 50°C, 50 rpm for 30 min reduced volatile off‐flavors, enhancing sensory appeal (Andaç et al. 2024). Blanching at 100°C for 3 min by immersing in boiling water deactivated lipoxygenase (LOX) in the milk (Andaç et al. 2024).	Pasteurization at 85°C for 2 min influenced protein solubility and ensured microbial stability (Vogelsang‐O’Vogelsang‐O'Dwyer et al. 2022). Cooking the seeds for 30 min at 100°C and pasteurizing the milk for 1 min at 100°C in a pressure cooker reduced the beany flavor (Lopes et al. 2020).
**Physical structure modification**	The addition of 1.5% and 3.3% sunflower oil enhanced the whiteness index and improved the emulsions' colloidal stability by reducing the milk oil droplet sizes (Jeske et al. 2019). Homogenization at 900 bar pressure enhanced stability and solubility by reducing the particle size by over 100‐fold (Jeske et al. 2019).	Milling the seeds for 4 min at 20,500 rpm, then colloidal milling at 70 rpm for 15 min, refined the mixture texture by creating a uniform suspension (Duarte et al. 2022).	Milling seeds in a blender at high speed for 3 min produced slurries, which eased separation in the milk production (Yan et al. 2024). Stabilizing agents such as locust bean gum, pectin, and guar gum (0.5%, 0.4%, 0.5%), enhanced stability, ensured a uniform texture, and reduced phase separation during storage of the pea milk (Kulczyk et al. 2023).	High‐pressure homogenization of 180 to 780 bar resulted in smaller particle size and greater stability of the lupin milk; pressure of 780 bar gave the most stable product (Vogelsang‐O'Dwyer et al. 2021).
**Component isolation and separation**	Filtration through a sieve with a 0.1 mm mesh size ensured a smoother and more uniform texture by removing larger particulates (Cichońska et al. 2023). Lentil protein isolates, containing 93.7% protein, enhanced the emulsion's structural integrity, making it ideal for plant‐based milk substitutes (Jeske et al. 2019).	Enzymolysis using papain at 75.0 U/g protein, α‐amylase at 69.0 U/g starch, and β‐glucosidase at 11.0 U/g flour converted isoflavone glucosides into more bioavailable aglycones, enhancing the milk's bioactivity (X. Zhang et al. 2022).	Filtration using a two‐layer gauze and centrifuging at 4°C for 15 min at 3000 rpm enhances milk texture to obtain smooth supernatants (Yan et al. 2024). Filtration from tangential microfiltration retains biochemical components, reducing nutrient content, but is more palatable (Aurèle et al. 2016).	Lupin protein isolates were used as they showed greater emulsion stability compared to dairy (Vogelsang‐O'Dwyer et al. 2022).

Soaking is a pre‐processing step for pulse seeds performed in an acidic, neutral, or alkaline soaking medium to reduce seed hardness for easy dehulling, reduce the alkaloid content, and reduce antinutrients as they leach out into the soaking medium (Manickavasagan and Thirunathan 2020). Pulses can be soaked for 12 to 24 h to achieve different outcomes depending on the variety and intended end use (Munthali et al. 2022; Locali‐Pereira et al. 2023). Duarte et al. (2022) found that soaking chickpea seeds in water for 16 h effectively reduced α‐oligosaccharides associated with digestive discomfort, while Lopes et al. (2020) found that soaking lupin seeds for 16 h decreased the bitterness. Andaç et al. (2024) reported an increased concentration of volatile compounds when pea seeds were soaked in alkaline water with a pH = 9 for 1 h after blanching in boiling water. Soaking can be combined with other processing techniques, like germination.

Germination, when initiated after soaking, can increase the simple sugar of pulses through the activation of amylolytic enzymes by decreasing the pulse seed's starch content (Cichońska et al. 2023). Germination is usually carried out under controlled temperature, humidity, and time conditions. Lopes et al. (2020) reported that germination can reduce complex carbohydrates such as the oligosaccharide content and is effective in reducing the anti‐nutritional factors causing the beany flavor in pulses due to phytate contents. The authors argued that germination can enhance the nutritional profile of pulse beverages by increasing protein bioavailability. Furthermore, Cichońska et al. (2024) studies on germinating lentils at about 25°C for 72 h at 85% humidity were found to increase fiber content through hydrating the polysaccharides, which are found in the seed's cotyledon cell walls. The authors reported that germination can enhance the health‐promoting benefits of pulse milk as dietary fiber present in food can lead to slower food absorption and enhanced satiety. In addition to germination, other processing techniques, such as blanching, can influence pulse milk quality.

Blanching is a processing technique used in vegetable processing to inactivate enzymes to ensure their nutritional value and original color are preserved (Saini et al. 2023). Conventional blanching, such as steam or boiling water blanching, has been reported to affect bioactive compound retention, leading to nutritional loss, hence the increased use of microwave blanching in food processing (Saini et al. 2023; Boateng 2024). Lipoxygenase enzyme was inactivated by blanching pea seeds in boiling water for 3 min in a study reported by Andaç et al. (2024). Cooking is a technique used to reduce the beany flavor in pulses, as it minimizes the presence of unpleasant volatile compounds (Duarte et al. 2022).

Pasteurization for a short or long time at different temperatures between 75°C and 95°C on the developed pulse milk aims to inactivate pathogenic microorganisms, ensure microbial stability, and preserve the nutritional content (Vogelsang‐O'Dwyer et al. 2022). Sterilization is a processing technique involving higher temperatures of about 121°C under high pressure to eliminate all viable microorganisms, including spores, which can extend milk shelf life (Cichońska et al. 2023). Sterilization ensures the safety of the milk after pre‐processing steps like milling, preventing final product contamination. Milling the pulses breaks down the seeds into smaller particles, improving the texture and creating uniform suspension by high‐speed milling, which can facilitate milk extraction and filtration (Duarte et al. 2022). Stabilizing involves the addition of hydrocolloids, emulsifiers, or other agents to prevent phase separation, reduce sedimentation, and ensure consistent texture throughout the shelf life of plant‐based milk. Additives such as guar gum, locust bean gum, and pectin (0.5%, 0.5%, 0.4%) were used to maintain a uniform suspension and enhance the viscosity of pea milk (Kulczyk et al. 2023).

The addition of oil in processing lentil milk, such as sunflower oil, enhanced emulsions through droplet size reduction and sensory appeal of the milk (Jeske et al. 2019). The stabilizing agents in pea milk prevented syneresis and reduced phase separation (Kulczyk et al. 2023). Pulses have low‐fat content, leading to colloidal instability; a combination of these techniques could offer synergistic effects. Stabilizers can prevent separation, syneresis, and sedimentation, which are common in pulse milk development, to improve sensory appeal and increase consumer acceptability (Asase and Glukhareva 2024). The product development of a stable pulse milk, combining different processing techniques, can increase market readiness. Patterson et al. (2017) studied and suggested that reducing the antinutrients in pulses depends on various factors, including pulse cultivar, growing conditions, and the combination of different processing techniques, such as soaking, thermal treatments, microwave heating, and irradiation. However, thermal treatments were more effective than other processing techniques.

Researchers have explored novel technologies such as ultrasound, high hydrostatic pressure, and UV radiation to develop plant‐based milk with extended shelf life (Sim et al. 2020; Sarangapany et al. 2022). Sim et al. (2020) reported the viscosity of yogurt made from lentil, chickpea, and pea protein isolates treated with high hydrostatic pressure to have gel strengths comparable to that of commercial Greek yogurt. The authors also mentioned some concerns, such as the inactivation of probiotic cultures under high pressures, recommending low pressure, and optimizing flavor for sensory quality. While these novel technologies have been mainly applied to popular plant‐based milks, such as soy, almond, and oat milk, they create an area of interest for researchers to explore in pulse milk product development. The implications of applying novel technologies, such as microwave and cold plasma treatment on pulse proteins and analyzing their pros and cons, have been reviewed by Verma et al. (2025).

Cream color and milky flavor are some of the attributes associated with positive drivers for eighteen plant‐based milk sensory evaluations studied by Jaeger et al. (2024). From the study results, the authors stated that pink and white colors in the analyzed milk were penalized. At the same time, earthy and beany flavors are strongly disliked product characteristics that negatively impact sensory liking for the products. Bonke et al. (2020), in a study involving lentil and pea milk, reported visible particles and a grainy appearance, giving the samples a sticky mouthfeel, which was likely caused by incomplete enzyme hydrolysis of starch in the seeds. A study on lentils on the addition of raspberry fruits to mask the beany flavor was reported by Cichońska et al. (2024) to enhance flavor and overall liking of the product developed. For lentil milk, a combination of high‐pressure homogenization and the inclusion of oil in the processing steps improved the stability and whiteness index, hence increasing the sensory liking of the product (Jeske et al. 2019). Pea milk phase separation was reduced during storage using gums as stabilizing agents to enhance stability, increasing appearance liking (Kulczyk et al. 2023).

### 3.2 AI Approaches in Optimizing Processing Parameters

Food processing in the era of current and emerging technology under the fourth industrial revolution has introduced new levels of automation involving big data, robotics, and AI (Hassoun et al. 2023). AI enables machines to imitate human cognitive processes and perform tasks that require human intelligence (Mavani et al. 2022). Machine learning can use algorithms to recognize occurring patterns and generate predictions in a study (Popenici and Kerr 2017; Armand et al. 2024). The food industry is applying AI to shorten product development time from years to months, which can save money and time. NotCo's Giuseppe platform creates plant‐based alternatives by analysis of ingredients at a molecular level (Buss 2023). These examples highlight how AI revolutionizes food product development, fosters innovation, and showcases how consumers' needs can be satisfied.

By integrating sensory data into machine learning models, large volumes of multidimensional data from sensory panels, chemical analyses, and instrumental measurements can accurately predict sensory outcomes (Freire et al. 2024). Several published studies have been conducted on machine‐learning applications of dairy milk and other food products, but none on AI applications in pulse milk product development. Çelik (2022) applied machine learning algorithms to model milk quality, providing high accuracy in determining the sensory quality of dairy products. Cortez et al. (2009) employed machine learning algorithms to predict the sensory quality of white and red wines based on their physicochemical characteristics, enabling a data‐driven approach to understanding and optimizing wine quality attributes.

## 4 Impact of Marketing Variables on Consumer Perception of Pulse Milk

### 4.1 Marketing Strategies for Pulse Products and Their Impact on Pulse Milk

The discussion in this section draws from broader research on marketing strategies, as there are no direct studies on the consumer perception of pulse milk identified. The limited availability may be due to pulse milk being a relatively new product category that is underexplored compared to other plant‐based milk alternatives. Therefore, this section highlights general insights that could be applied to pulse milk to gain market attention.

Factors influencing consumers to purchase pulse milk alternatives are crucial when considering marketing strategies. Involvements, regarded as how a consumer perceives a product based on their interest, values, and inherent needs, could be applied as a marketing variable to increase purchase decisions (Chen et al. 2022). According to the U.S. Department of Agriculture, in 2024, the crop value of pulses, specifically dry beans, peas, lentils, and chickpeas in the United States, reached around 2.17 billion USD (Davis et al. 2024). Kumar et al. (2023) argued that the adoption of enhanced farming methods and pulse varieties has contributed to an increase in total pulse crop production. Pulses are naturally gluten‐free, and consumer demand for pulse products perceived as healthier snacks increases with consumer demand for allergy‐free food (Bond 2017). With the current rise in demand for nutritious foods, there is a chance of mislabeling, which can negatively influence consumer perception and trust in the food industry (Kehagia et al. 2007). Chang and Chen (2022) described “clean label” as the presence or absence of certain additives or preservatives used in foods. The authors argued that mislabeling could involve using words like “natural” or “clean label” or not clearly disclosing allergens without required certifications. The study also mentioned that 46% of Americans' purchasing decisions are influenced by simple food labeling. Shaping consumer perception to increase acceptance of pulse products can be an effective marketing strategy.

Pulses have been associated with more negative implicit attitudes than cereals because of dislike of the sensory attributes of pulses, perception of pulses as primarily suited for vegetarian diets, and difficulty in preparation, leading to the low consumption of these pulse‐based products (Melendrez‐Ruiz et al. 2023). Prytulska et al. (2021) reported in a study in Ukraine that 38% consumers mentioned taste and 21% mentioned price as an important factor for their purchasing decision for plant‐based milk. Plant‐based products tend to be priced slightly higher, which affects their marketing strategies because price is a key driver of consumer purchasing intent (Lehmann et al. 2025). The marketing power for plant‐based products, including pulses, may increase with price reduction.

Chrysochou et al. (2010) stated that consumers can easily switch to plant‐based milk alternatives as they are perceived as healthier through a branding market strategy that trusts and resonates with them. The study argued that health claims on food products can be used to appeal to consumers; for example, excluding sugar can be used to promote specific plant‐based milk categories. Lactose intolerance, flexitarian practices, and environmental concerns are reasons for the increase in global consumption of plant‐based milk (Jaeger and Giacalone 2021). According to Tso et al. (2020), the environmental and health benefits rather than the taste of plant‐based milk are among the reasons consumers switch from dairy milk. Ethical, environmental, and social concerns have been a growing cause for international vegans and vegetarians (Leitzmann 2014).

### 4.2 Psychological and Emotional Appeal in Marketing

Marketing variables, such as customer experience, pricing, brand reputation, emotions, perceived value, and loyalty, are essential in shaping consumer perception of a product (Santos et al. 2020). Marketing theories primarily focus on consumer responses through understanding the attitudes, reactions, traits, and decision‐making processes of individuals (Childs et al. 2024). Consumer attitudes toward a product can stem from experience, market adverts, or social media influence. Claims on the front or back of food packages can be marketing, nutritional, health‐related, or short phrases printed and used as a marketing tool to inform consumers about the benefits and ingredients (Steinhauser et al. 2019). Marketing claims like “small batch,” “locally made,” and “made with love” on product packaging can influence consumer purchase (Waehning et al. 2018). An attractive product package can serve as a marketing strategy to draw consumers' attention.

According to Kuvykaite et al. (2009), attractive product packaging is a necessity in marketing, as it could be a medium to attract a consumer's interest when considering the purchase of a product or during impulsive buying. Zhao et al. (2021) study findings argued that consumers might choose to purchase a cheap local product over a product with attractive packaging because of its high price. The authors reported that pricing and information on product packaging play a crucial role in consumer buying behavior and satisfaction. Consumers can be attracted to products with detailed labeling. Consumers who are committed to sustainability issues would prefer purchasing a product with recyclable packaging that would reduce environmental pollution and reduce resource use, influencing their food choices (Mancuso et al. 2021). Consumers' increasing awareness of sustainable packaging has drawn the attention of companies and policymakers to critically link food waste and packaging to the Sustainable Development Goals, increasing recyclable behaviors (Boz et al. 2020). According to Mahajan et al. (2023), the food industry and researchers are continuously exploring eco‐friendly materials for consumer products, deviating from non‐biodegradable packaging materials that lead to waste disposal that deteriorates our environment.

Adverts, educational posters, and informational campaigns are some of the strategies that can enlighten consumers about plant‐based milk made from pulses. Marketers can leverage strategies that promote pulse crops through campaigns and advertisements designed to reinforce positive associations through communication with influential chefs and culinary figures, encouraging the addition of pulses to their menus and consumers’ diets. Companies can create marketing strategies that resonate with sustainability and health‐conscious consumers who have changing dietary habits, making appeals for increased product purchases.

### 4.3 AI Applications in Marketing and Consumer Segmentation

Machine learning algorithms can analyze diverse collections of data such as online reviews, information shared on social media, and consumer transactions, delivering insights on marketing models to understand consumer preferences (Modak et al. 2024). Technology such as AI can help businesses create future marketing campaigns and strategies that promote their products, reducing the complex decisions that marketers face when reaching consumers (Dumitriu and Popescu 2020). According to Herhausen et al. (2024), companies' staff can make consumer‐friendly marketing decisions by using AI to generate predictions on offers that would target intended customers. Digital marketing can help determine the content that attracts consumers, increasing the likelihood of subscription through the application of AI (Nair and Gupta 2021). According to Haleem et al. (2022), marketing goals can be achieved by guiding consumers using intelligent email marketing and AI chatbots in a direction that aligns with the marketing goal. The study discusses how mind‐numbing advertising that does not resonate with consumers and wastes time can be avoided using analyzed datasets.

According to a report by Deloitte, employing machine learning models in customer engagement reached approximately 10% of customers and generated over $100 million in revenue (Deloitte 2022). Overall, AI enhances decision‐making, optimizes campaigns, improves efficiency, and enables more targeted strategies as a powerful, evolving technology. To improve productivity and enhance innovation, embracing AI in digital marketing can be important in the coming years (Sharma et al. 2024). According to Canvas Business Model (2024), 20% of plant‐based milk sales made by Ripple Foods came from targeting health‐conscious specialty retailers. This example illustrates how a marketing strategy can establish a promising business model in a competitive landscape.

## 5 Demographic Variables Affecting Consumer Perception of Pulse Milk

### 5.1 Key Demographic Factors That Can Impact Pulse Milk Acceptance

Demographic variables, such as culture, education, income, gender, and age, are popular with marketers as they provide links to consumer needs, help estimate market size, and are easy to measure (Kotler and Keller 2015). Social and economic factors influence the food choices consumers make during purchase. As the food sector becomes unique and increasingly different, food product developers face challenges predicting consumer food choices (Grasso et al. 2023). Understanding consumer perceptions of different food products and their integration into their diets by demographics has become essential to product success (Bogueva 2024).

Age is an influential demographic variable in consumers’ food choices because different age groups have different nutritional needs, culinary skills, incomes, lifestyles, and other confounding variables. The older generation is more inclined toward a food category because they are more conservative and patriotic (Lugioyo 2022). However, natural, organic, lower salt and sugar content foods are drawn towards older adults in a desire to address health issues that come with age, and it impacts consumer food choices (Rai et al. 2023). The younger generation's perceptions of foods are instantaneous, as they are likely to taste a new food product, try a different brand, and focus less on prices than older consumers (Helm and Landschulze 2013).

In terms of age's influence on pulse consumption, Winham et al. (2020) studied pulse and pulse food item consumption amongst college‐aged students in the United States. They reported that 64% had never tried to cook pulses, while pulse‐based snacks, salad, and hummus are eaten often or once a month by these students. Males aged between 31 and 70 are more likely to consume pulses, while both male and female younger adults aged between 19 and 31 years are less likely to be pulse consumers (Mitchell et al. 2021). Compared to others, younger individuals perceived more barriers to pulse consumption, citing low levels of education and financial difficulties as primary reasons (Kuosmanen et al. 2023).

Gender tends to influence consumers' buying decision process (Suvadarshini and Rout 2024). In making purchase decisions and eating habits, it is evident that each gender has different preferences for food products. Manippa et al. (2017) argued that females, being more concerned about weight gain, tend to choose diets that are higher in dietary fiber and include more vegetables and fruits, while consuming fewer fatty foods, compared to males. The study conducted in Italy also stated that males tend to consume food in larger quantities to satisfy their appetites while being concerned about fatty foods or gaining weight. Gender differences in food choices are also evident in consumer perception of brand loyalty. Studies have shown that women purchase a brand known for high quality, are loyal to it, and influence men to switch brands, purchasing the products they choose (Lugioyo 2022).

Kuosmanen et al. (2023) earlier studies in Finland had shown that women with higher education and interest in eating healthy are the typical pulse consumers, while men with higher income and disinterest in eating healthy prefer animal meat as a source of protein. The study also argued that men were less likely than women to see not knowing how to cook pulses as a barrier to pulse consumption. Pulses are food products known to contain less fat and have high dietary fiber, which could be a factor showing why studies have reported women to be more likely to consume pulse products than men. Consumer perception of a product type varies based on emotional involvement, which may differ by gender, influencing their purchase intention (Baek and Kim 2022).

Income level can determine access to a variety of quality food, which directly impacts the buying decision and plays a vital role in food choices made by consumers (Ahmeti 2022). Higher‐earning individuals will purchase high‐quality food options, such as organic and sustainably produced products. At the same time, lower‐income earners prioritize affordability because they might have limited choices (Attree 2005). Arganini et al. (2012) reported that consumers would choose to go for quantity over the quality of a food product because of income level and would not consider the nutritional content over the affordability of that product. Financial, social, economic, and cultural factors are involved in why people eat what they do (Kuosmanen et al. 2023), which could also shape consumer perception of pulse milk. Barrientos‐De La Rosa et al. (2023) reported that individuals with low‐income levels and larger household sizes are more likely to consume pulses.

Cultural background, health concerns, and sensory preferences are consumer perceptions that shape their attitudes, whether negative or positive, on the consumption behavior toward pulses (Melendrez‐Ruiz et al. 2023). The influence of tradition, religion, and ethnicity are cultural factors that shape individuals' diets, such as different cultural groups' unique dietary preferences, taboos, and beliefs, which hugely affect how they perceive food choices (Monterrosa et al. 2020). Culturally, consuming pulses preserves and revitalizes traditional dishes, promoting the shift to plant‐based diets that support environmental sustainability and reduce ecological impact, water use, and carbon footprint (Barrientos‐De La Rosa et al. 2023). Plant‐based milk consumption may be challenged in countries with more traditional ties to the dairy industry, such as Italy, which has a cultural attachment to animal milk products (Irene 2023). However, the consumption of plant‐based diets in regions with heightened health awareness and environmental concerns has seen a paradigm shift in the adoption of food products from plants (Shah and Joshi 2024).

### 5.2 AI‐Driven Demographic Insights

AI can analyze consumer preferences based on broad demographic variables like income, age, and gender to identify nuanced patterns in complicated consumer data (Madanchian 2024). Digital marketers use AI to analyze demographic variables, identify opportunities, automate tasks such as content optimization, and target specific customers of different age groups and cultures (Yaiprasert and Hidayanto 2023). Consumer income level can play a role when food companies target specific demographics. Economic conditions sometimes fluctuate, and marketers must be agile in creating campaigns that respond to these fluctuations, as pricing is a key determinant of consumers' choice to purchase a food product (Khamoushi 2024). AI can identify an opportunity to develop new pulse milk recipes with different flavors that target specific demographics and new customer segments (Yaiprasert and Hidayanto 2023). Consumer behavior based on gender or age and marketing strategies can interact dynamically, yielding AI insights when analyzed to predict consumer perception in the future (Madanchian 2024).

AI‐driven strategies can consistently and continuously analyze consumer interaction data and allow for strategy adjustment to target individuals from different income levels as the economy changes. Applying AI to different demographic data can eliminate the one‐size‐fits‐all approach, create personalized messages, and maximize return on investment (Khamoushi 2024). Budget limits and preferences have altered purchases for items and payments for services individuals utilize to enhance their quality of life. The application of AI has transformed complicated consumer preferences and behavior into insights based on demographic analytics. AI and machine learning have grown enormously in the last decade, primarily because they are used to create technical conclusions and solve issues (Tachie et al. 2023). However, despite the effectiveness of AI in analyzing demographic variables for consumer preferences and targeted advertising, it still faces limitations in creating emotional connections and personalized messages (Khamoushi 2024).

There are limited studies on demographic insights into pulse milk research. However, AI has been applied to different areas, focusing on demographics. In a study by Lee et al. (2021), an AI model was used to analyze 1.4 million restaurant reviews, predicting how the industry can attract customers based on attributes like location, reviewer, and linguistic content. The study stated that the application of machine learning models can enhance customers' perceptions and decisions to purchase food from the restaurant. Kazama et al. (2018) reported an AI model that can transform food recipes from different cultures into a new regional style, such as from Japanese to French recipe style. The authors stated that food companies and chefs can create recipes tailored to the growing cultural diversity.

## 6 Traditional Versus AI‐Facilitated Research

### 6.1 Challenges in Applying AI in Pulse Product Development—Scalability, Biases, and Readiness to Adoption

Developing new food products from pulses can encounter challenges in product optimization and scalability. Traditional research offers both quantitative and qualitative insights by capturing emotional, cultural, and psychological consumer preferences (Attree 2005). However, data interpretation in traditional research heavily depends on statistical methods, which may overlook complex, nonlinear relationships between variables (Slob et al. 2021). AI models excel at analyzing high‐volume datasets such as sensory responses, chemical compositions, and processing parameters to predict optimal product formulations (Freire et al. 2024). Nevertheless, AI algorithms require vast, high‐quality datasets to make accurate predictions. The issue that can arise in applying AI in pulse milk product development is scalability, as existing datasets have been collected under only a limited selection of processing conditions.

Armand et al. (2024) stated that the lack of datasets that represent different demographics could create algorithmic bias in developing AI models for food and nutrition research, causing bias and model performance disparities that could limit their generalizability. The limitation may be partially related to having non‐random samples that might systematically differ from larger groups of interest. Furthermore, Hassoun et al. (2022) stated that the food industry is slower than other sectors and at an early stage in adopting and implementing new technologies such as AI due to some challenges like lack of technical skills and privacy issues. The author suggests that financial investments and more research focused on a digital food future can help overcome these obstacles in the food industry.

The emergence of ChatGPT, Gemini, and DeepSeek, which are generative AIs, has led individuals to contemplate whether the use of AI is replacing traditional research. There are two different types of AI, namely non‐generative AI, which predicts, optimizes, and discovers, while generative AI produces and creates new data (Kuhl 2025). This concise review discusses overall insights on traditional and AI research, while other authors have compared both. Kuhl (2025) exhaustively discussed the traditional food development approaches in contrast and combination with how AI can be used to predict and accelerate product development. Limited studies exist on AI applications in pulse milk development.

Freire et al. (2024) reviewed machine learning, a subset of AI, and its application on daily milk products, including collecting data, cleaning data, testing, and validation of the model to predict outcomes. When combined with experimental sensory and flavor chemistry studies, AI can help identify optimal processing parameters. Schreurs et al. (2024) reported that machine learning models were used to predict the flavor and consumer perception of 250 Belgian beers based on sensory analyses and chemical properties. The study, which combined traditional research and AI, showed that given the complexity of food flavors, using only traditional research that applied basic statistical tools might not be suitable for accurate predictions. FLAVOUR‐AI is a tool launched by Wageningen Food and Biobased Research that leverages AI to improve the flavor of plant‐based products, while the research is still in the early phases, it could be beneficial. Future studies need to educate stakeholders that AI is not replacing human or traditional research, but enhancing and reducing time when combined. AI can be applied in product development to optimize datasets and predict consumer preferences. However, it is worth noting that AI cannot independently resolve sensory challenges as it requires input datasets that can be acquired from traditional research, such as sensory and physicochemical data. To the best of our knowledge, there is no published literature demonstrating solely the application of AI to solving flavor issues in plant‐based milk.

### 6.2 Limitations of AI in Accounting for Emotional and Psychological Factors

According to Xia et al. (2024), consumer trust in food innovation using AI is essential, as the quality, nutritional value, and taste of AI foods impact consumer satisfaction in terms of cognitive experience and emotional reliability. Food neophobia, a reluctance to try unfamiliar food (Su et al. 2023), may not be captured by the AI model, impacting sensory data analysis. Consumers may believe that the food industry's use of new technologies prioritizes profit over the health of its customers, having thoughts that the application contradicts the production of nutritious and tasty food (Siegrist and Hartmann 2020; Meijer et al. 2021). Educating consumers about AI and creating awareness of the benefits of new technologies can increase their trust and shift the consumer's mindset that producers only benefit from its adoption (Meijer et al. 2021).

## 7 Knowledge Gaps and Future Directions

The plant‐based milk market is dominated by soy, almond, oat, and coconut milk, which are found on shelves in big grocery stores. Pea milk is the most available commercial pulse milk, while less common products such as chickpea and Bambara milk are available only through a limited market space. Lentils and lupins are pulse crops that can also be used in producing pulse milk, yet they remain underexplored. The commercialization of pulse milk remains limited by brands despite pulse crops' environmental and nutritional benefits. The inclusion of pulses in the milk market can diversify the plant‐based milk options and increase the cultivation of pulses, mitigating greenhouse gas emissions and reducing food production's environmental impact (Hossain et al. 2016).

One of pulse milk's market challenges is overcoming the sensory barriers, mainly related to flavor, texture, and appearance (Roland et al. 2017). Advanced processing techniques, such as enzymatic treatments and homogenization, can improve the sensory quality of pulse milk (Vogelsang‐O'Dwyer et al. 2021; Zhang et al. 2022). Despite these advancements, limited commercialization indicates that pulses are still underexplored as milk products, and further research and development are needed. Consumers with sustainability and environmental health consciousness have a higher tendency to consume plant‐based food products (Su et al. 2023). However, sustainability remains the last, while taste is the first main driver for consumers to purchase a food product (IFIC 2020).

Understanding consumer perceptions is crucial for effectively marketing pulse milk to environmentally conscious consumers. There are various studies on the environmental and health benefits of pulse crops. However, 61% of consumers displayed knowledge gaps on the climate benefits of pulses in a study conducted in Poland (Szczebyło et al. 2020). Kuosmanen et al. (2023) reported that in a study in Finland, about 41% of consumers do not like the pulse taste, and Cichońska et al. (2024) reported beany flavors, attributed to lipoxygenase activity, and a chalky texture resulting from insoluble large particles present consumption challenges. Advanced processing technologies, AI‐driven optimization, targeted marketing, and education can enhance pulse milk's quality, consumer perception, and market adoption.

## 8 Conclusion

The rate at which the plant‐based milk market continues to grow shows that companies can benefit from product innovations. Pulse milk offers an alternative to dairy, as studies have shown that milk can be developed from lentils, lupins, chickpeas, and peas, which are eco‐friendly and nutritious. Taste remains the primary factor for the limited commercialization and consumer acceptance of pulse milk. Sensory studies on pulse milk have reported beany, grassy, and earthy notes as the most common negative attributes noted by consumers. However, a combination of advanced processing techniques such as high‐pressure homogenization, pressure cooking, and flavoring can mask the off‐flavor and enhance the taste of pulse milk to increase consumer acceptance. A combination of multiple factors, including processing and effective marketing strategies targeting different demographics used as an indicator during product development, can influence consumer perception of pulse milk. AI usage in optimizing multiple variables can be integrated with traditional research methods to overcome its scalability, bias, and adoption.

This review identifies three main methods to increase pulse milk consumption. First, advanced processing techniques can overcome sensory barriers. Second, tailored marketing strategies can target different demographics to encourage consumption. Third, combining AI with traditional research can capture complex insights in both processing and marketing to enhance consumer acceptance of pulse milk. Further research is needed to explore optimal formulation and processing to enhance the nutritional content and sensory appeal of pulse milk. Marketing intervention studies investigating pulse milk are still limited and should be explored to increase consumer awareness of pulse milk.

## Author Contributions

**Chidimma Ifeh**: conceptualization, methodology, data curation, investigation, visualization, writing – original draft, writing – review and editing, project administration. **Bradley Whitaker**: writing – review and editing, supervision. **Neda Nazemi**: writing – review and editing, supervision. **Mark Greenwood**: writing – review and editing, supervision. **Mary Miles**: writing – review and editing, supervision. **Wan‐Yuan Kuo**: conceptualization, methodology, investigation, supervision, writing – review and editing, visualization.

## Conflicts of Interest

The authors declare no conflicts of interest.
